# Bilateral Maxillary Dentigerous Cysts in a Nonsyndromic Child: A Rare Presentation and Review of the Literature

**DOI:** 10.1155/2018/7583082

**Published:** 2018-04-15

**Authors:** Rakshit Vijay Khandeparker, Purva Vijay Khandeparker, Anirudha Virginkar, Kiran Savant

**Affiliations:** ^1^Department of Oral and Maxillofacial Surgery, Goa Dental College and Hospital, Bambolim, Goa, India; ^2^ICU Horizon Hospital, Margao, Goa, India; ^3^Rejoice Aesthetic Centre, Bangalore, Karnataka, India

## Abstract

Dentigerous cysts represent the second most common odontogenic cysts of the jaws after radicular cysts and are usually associated with the crowns of unerupted permanent teeth and rarely deciduous teeth. They are usually solitary in their presentation. Multiple and bilateral dentigerous cysts are an extremely rare presentation in the absence of developmental syndromes or systemic diseases or the use of prescribed certain medications. We hereby present a case of a bilateral dentigerous cyst of the maxilla in a 10-year-old child involving the crowns of unerupted permanent second premolar on the right side and the unerupted permanent canine on the left side. An effort has also been made to review the existing literature on this entity and to stress the importance of radiographic and histopathological examinations in diagnosing such an entity.

## 1. Introduction

Dentigerous cyst (DC) is an epithelial-lined developmental cavity that encloses the crown of an unerupted tooth at the cementoenamel junction. It accounts for nearly 24% of all the true cysts in the jaws, thus being only the second most common to radicular cysts in the frequency of occurrence [1, 2]. The usual presentation is that of a solitary lesion; however, in the presence of developmental syndromes or systemic diseases or the concurrent use of certain drugs, bilateral or multiple lesions are seen to occur [3–6]. Having said that, the occurrence of the bilateral DC in the absence of any of these factors is an extremely rare occurrence. We hereby present a rare case of a bilateral DC of the maxilla in a nonsyndromic 10-year-old child. The authors believe that this is one of the only four cases to have presented in the maxilla bilaterally and therefore needs a special mention. In fact, the review of existing English literature and extensive search on PubMed database from 1943 to 2017 brought to light a total of 30 cases of the bilateral DC, 3 of which have been reported in the maxilla so far, while 24 have been reported in the mandible and the remaining 3 in both the maxilla and the mandible [2, 6]. These statistics only point towards the true rarity of the condition.

## 2. Case Presentation

**Figure 1:**
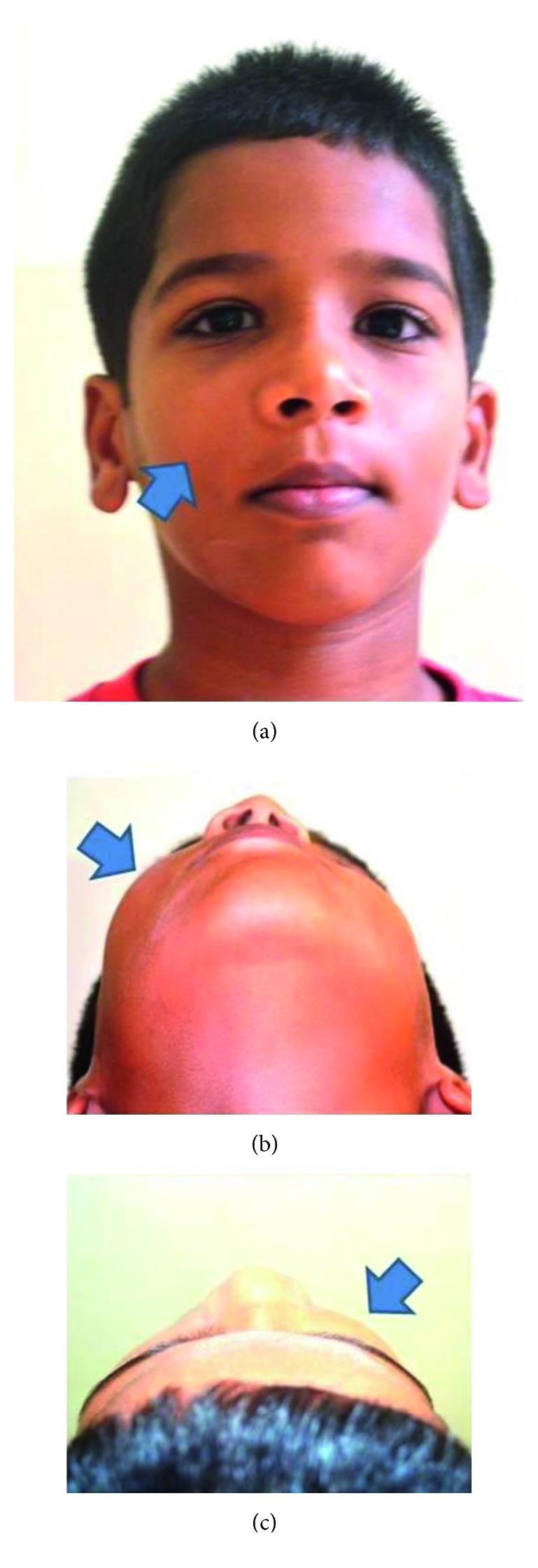
(a–c) Extraoral photographs with arrows depicting the facial swelling. (a) Frontal view. (b) Basal view. (c) Bird's eye view.

**Figure 2:**
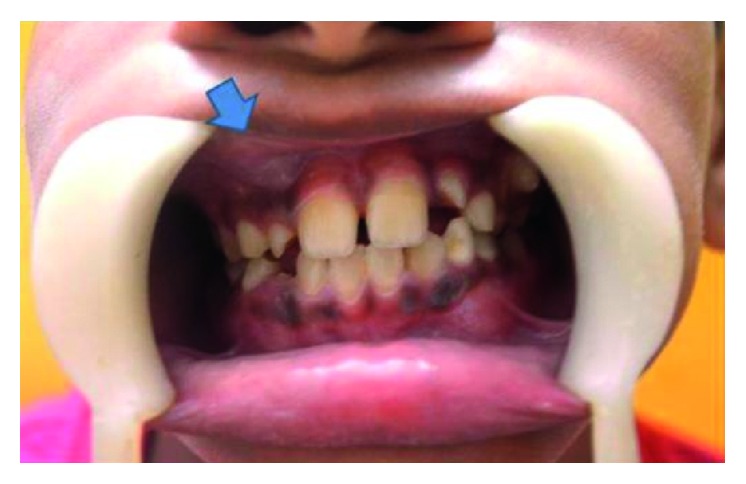
Intraoral photograph showing vestibular obliteration in relation to the right maxillary vestibule as depicted by an arrow.

**Figure 3:**
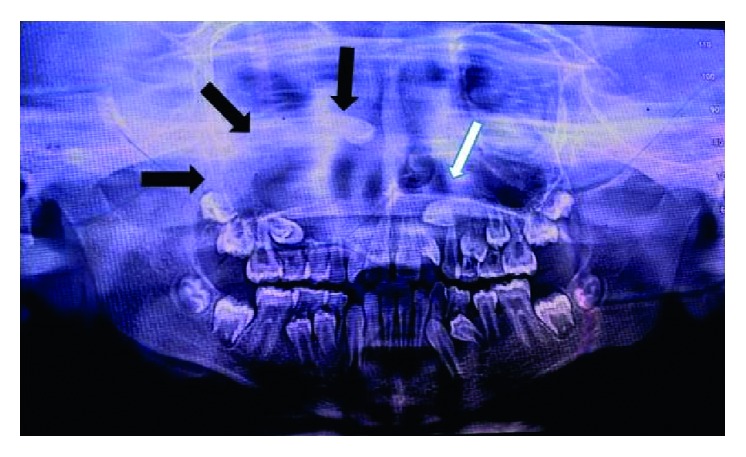
Panoramic radiograph showing bilateral unilocular radiolucencies depicted by black arrows on the right side and by a white arrow on the left side.

**Figure 4:**
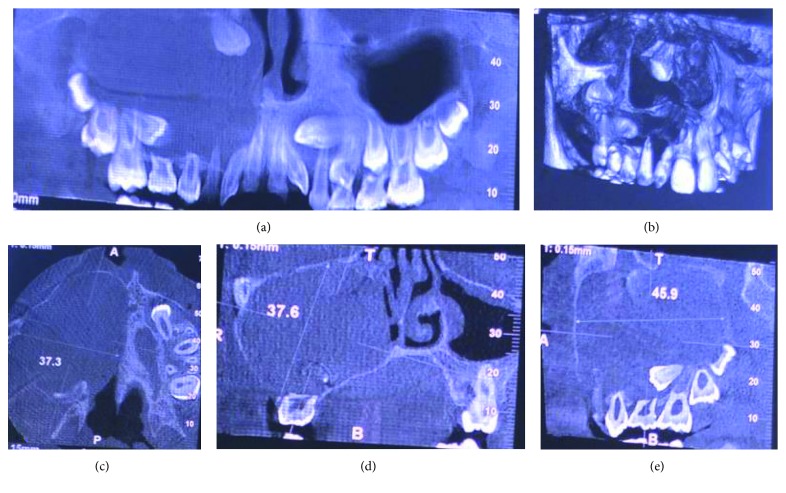
CBCT views with 3D reconstructed view showing more precisely the right maxillary radiolucency in relation to the crown of the unerupted permanent second premolar.

**Figure 5:**
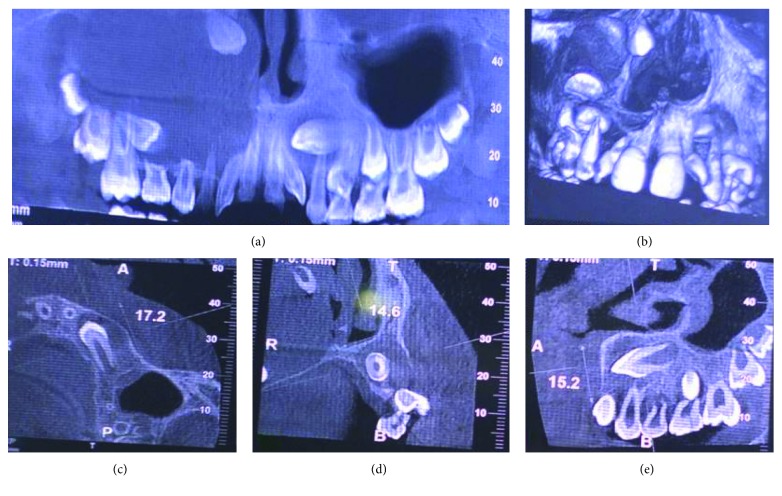
CBCT views with 3D reconstructed view showing the left maxillary radiolucency more precisely in relation to the unerupted permanent maxillary canine.

**Figure 6:**
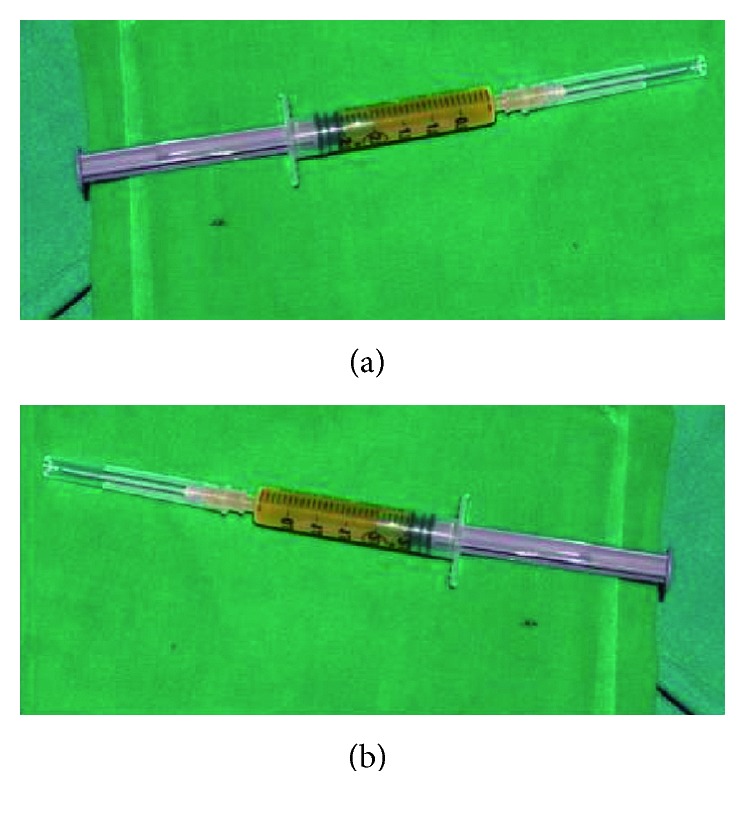
Aspiration of the cystic lesions revealing a straw-coloured fluid. (a) Aspirate from the right maxillary lesion. (b) Aspirate from the left maxillary lesion.

**Figure 7:**
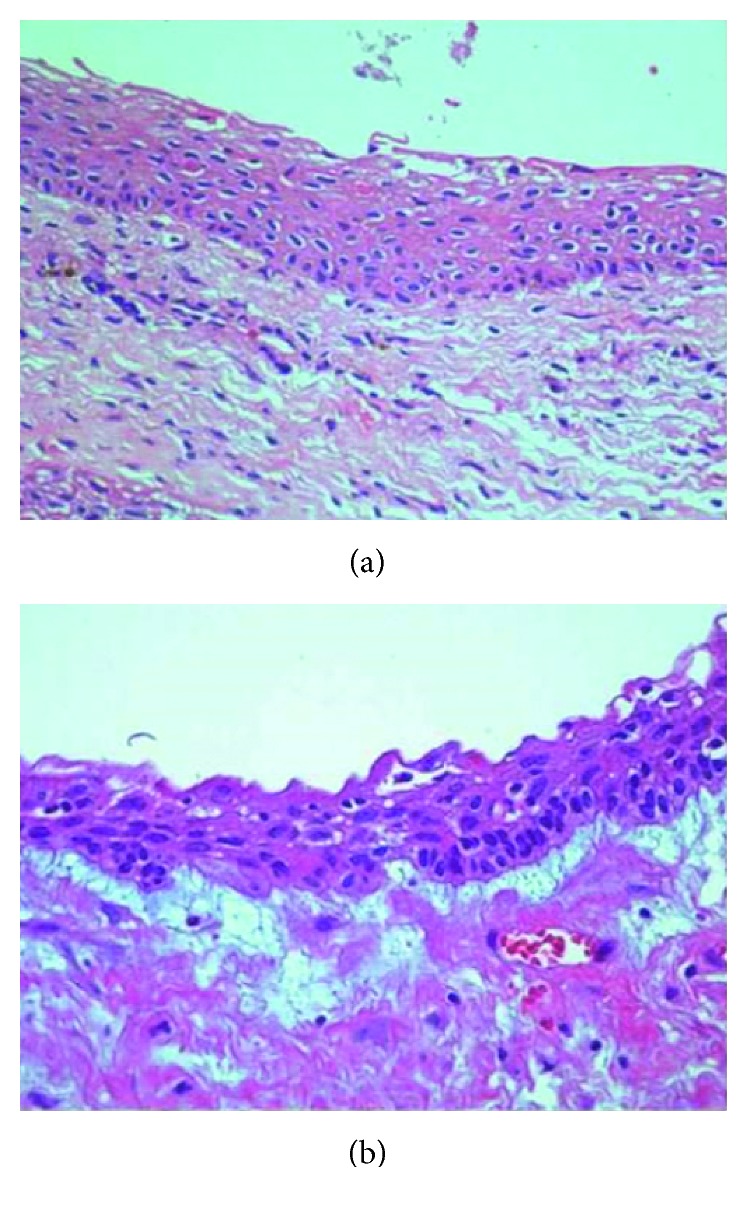
Histopathological examination of the bilateral maxillary lesions showing features of dentigerous cysts. (a) Histopathological picture of the right maxillary lesion. (b) Histopathological picture of the left maxillary lesion.

**Figure 8:**
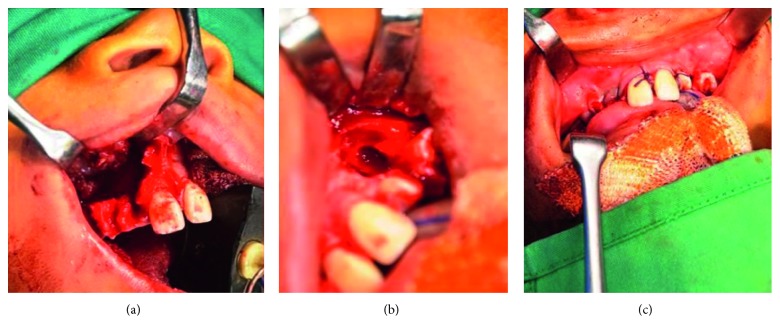
Intraoperative photographs of the patient. (a) Enucleation of the right maxillary lesion. (b) Enucleation of the left maxillary lesion. (c) Closure.

**Figure 9:**
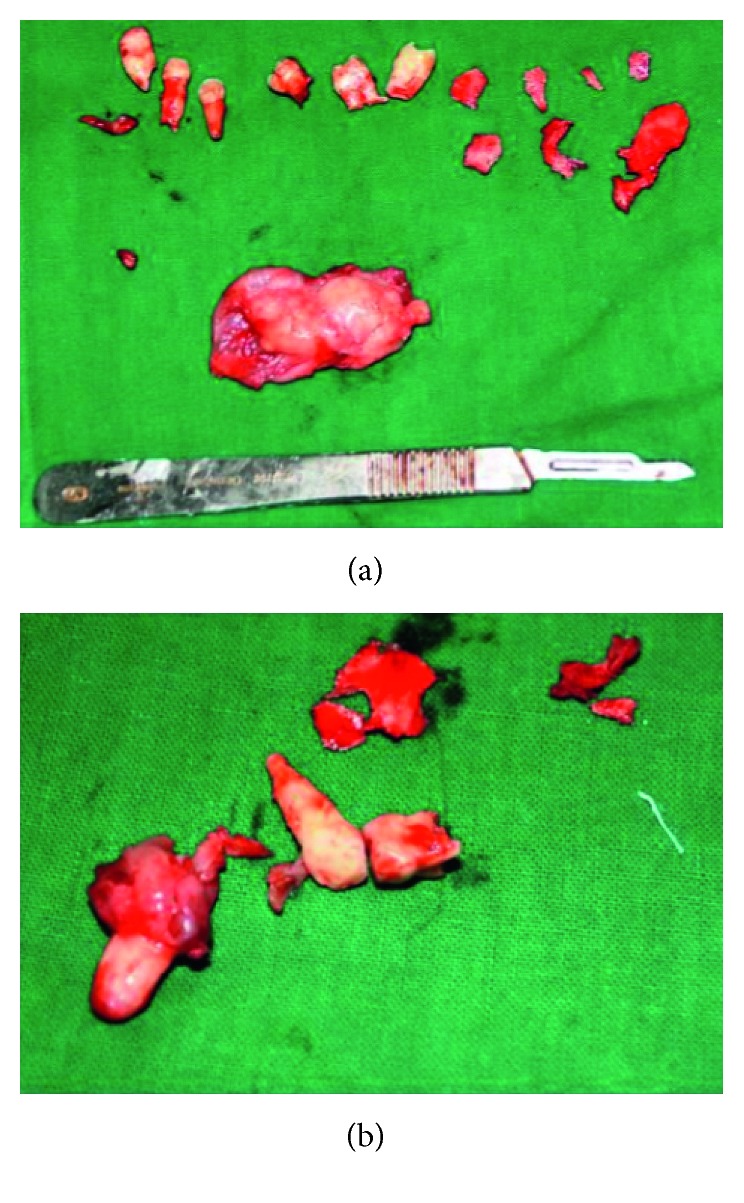
Specimens following enucleation submitted for histopathological examination. (a) The enucleated right specimen together with extraction of involved teeth. (b) The enucleated left specimen together with extraction of involved teeth.

A 10-year-old child reported to our unit with the chief complaint of swelling in relation to the right middle third of the face since one month. On extraoral examination, except for the facial symmetry on the right side and deviation of the nasal dorsum to the left side, no other relevant clinical findings were present (Figures 1(a)–1(c)). Intraoral examination revealed a mixed dentition with vestibular obliteration present from the maxillary right lateral incisor extending posteriorly towards the right maxillary permanent molar (Figure 2). The mucosa over the swelling appeared normal, and there was no evidence of discharge from the swelling. A panoramic radiograph was advised that revealed a well-defined unilocular radiolucent lesion with sclerotic margins measuring roughly 4 cm × 3 cm in greatest dimensions and arising from the cementoenamel junction of the unerupted right maxillary permanent second premolar and extending to involve the entire maxillary sinus with displacement of the unerupted right maxillary permanent canine towards the medial aspect of the orbital floor. There was radicular resorption seen in relation to the right maxillary deciduous canine and right maxillary deciduous second molar as well as in the right maxillary permanent first premolar. What was interesting to note was the presence of an occult well-defined unilocular radiolucent lesion with sclerotic margins in relation to the unerupted left maxillary permanent canine measuring roughly 1.5 × 1.5 cm in greatest dimensions (Figure 3). The extensive involvement of the right maxillary bone prompted us to perform advanced imaging in the form of cone beam computed tomography (CBCT) with three-dimensional reconstruction. The presence of bilateral maxillary lesions and the origin, size, bony destruction, expansion of the cortical plates, and involvement of the adjacent teeth were all confirmed using the axial, sagittal, and coronal views of CBCT (Figures 4(a)–4(e) and 5(a)–5(e)). The right maxillary lesion was extensive involving the entire maxillary bone with encroachment into the right nasal cavity and up to the right orbital floor (Figures 4(a)–4(e)). The aspiration of the bilateral lesions was attempted for the purpose of protein estimation which revealed a straw-coloured fluid with shiny cholesterol crystals with protein estimation values of 7.34 g/dl and 6.95 g/dl from the right and left lesions, respectively (Figure 6). A thorough systemic examination and laboratory testing ruled out the presence of any associated syndromes or systemic disease. We decided to carry out incisional biopsies of the bilateral lesions under general anesthesia to get a clearer picture of the pathology. Both specimens submitted for histopathological examination revealed a thin fibrous cystic wall lined by the 2-3 cell layer thick nonkeratinised stratified squamous epithelium. Rete pegs were absent, and the connective tissue showed mild inflammatory cell infiltrates. The subepithelial layers showed parallel bundles of collagen fibres at the periphery. These findings were consistent with the DC (Figures 7(a) and 7(b)). Under general anesthesia, enucleation of the bilateral cystic lesions was carried out together with extraction of the involved teeth, and closure was affected using 4-0 Vicryl sutures in a watertight manner (Figures 8(a)–8(c)). The specimens were submitted for histopathological examination that confirmed the diagnosis of the bilateral DC (Figures 9(a) and 9(b)). No dysplastic or metaplastic changes were noted when the entire specimen was examined histopathologically. Unfortunately, the patient was lost to follow-up almost immediately following the discharge, and therefore, no postoperative photographs or radiographs could be presented in this case to understand the treatment outcome. Nevertheless, considering the fact that bilateral DCs are rarely reported in the literature, this case deserves to be reported despite this minor pitfall.

## 3. Discussion

DCs represent benign odontogenic cysts associated with crowns of either unerupted or impacted permanent teeth or supernumerary teeth or odontomas but rarely deciduous teeth [3, 7]. They mostly present in the second or third decades of life and are rarely seen during childhood [3]. Seventy-five percent of the cases are seen to occur in the mandible. The substantial majority of DCs involve mandibular third molars followed by the maxillary permanent canine followed by mandibular premolars, maxillary third molars, and rarely maxillary premolars. In our case, the cysts were seen encircling the crowns of the unerupted right maxillary permanent second premolar and the permanent left canine. Studies have shown that the incidence of the DC involving the maxillary premolar was 2.7% as compared to 45.7% involving the mandibular third molar [2]. Mourshed stated that 1.44% of the impacted teeth undergo DC transformation, so DCs involving premolars are rare [8]. Daley et al. reported an incidence rate of 0.1–0.6%, whereas Shear found the incidence to be 1.5% [1, 2, 9].

The exact histogenesis of this entity is not clearly understood. It is seen to develop by accumulation of the fluid between the reduced enamel epithelium and enamel or within the enamel organ. The venous outflow is seen to get obstructed due to the pressure exerted by the empty tooth on an impacted tooth follicle, leading to rapid transudation of serum across capillary walls. This leads to the increase in hydrostatic pressure of the pooling fluid with resultant separation of the follicle from the crown with or without the reduced enamel epithelium. The development of the DC is also seen to be influenced by an intrafollicular spread of periapical infection from the deciduous tooth [4, 10].

DCs usually occur solitarily in most instances, and bilateral occurrence is an extremely rare finding. Bilateral and multiple DCs have been reported to occur in association with the number of syndromes or systemic diseases including basal-cell nevus syndrome, Gardner's syndrome, Maroteaux–Lamy syndrome (mucopolysaccharidosis type IV), cleidocranial dysplasia, and Klippel–Feil syndrome [3–6]. Sometimes, this entity is induced by prescribed medications like the combined use of cyclosporine A and calcium channel blockers [2, 4]. Pleomorphism in chromosome 1qh+ has also been reported with this condition [2, 4]. In our case, syndromic association or history of systemic medication was clearly ruled out through the absence of abnormal physical findings or laboratory results and through the absence of the use of any systemic medications.

A comprehensive search of PubMed and English literature could locate a total of 30 cases of nonsyndromic bilateral DC, 24 of which occurred in the mandible, 3 in the maxilla, and 3 in both the maxilla and the mandible [2, 6]. The age range for reported cases was seen to vary from 5 to 57 years, and the mandibular teeth were most frequently involved in the majority of the cases [2, 6]. The most common site was the mandibular third molar (11 cases), followed by the mandibular first molar (10 cases), maxillary third molar (2 cases), maxillary cuspid (2 cases), mandibular first premolar (2 cases), mandibular second premolar (1 case), mandibular central incisor (1 case), and maxillary central incisor (1 case) [6, 11, 12]. In our case, the age of the presentation was 10 years, and the cysts were seen to involve the right maxillary permanent second premolar and the left permanent canine. Only 3 cases of bilateral maxillary involvement have been reported so far in the literature. To the best of our knowledge, this is only the fourth case of the simultaneous presentation of the DC bilaterally in the maxilla. This finding points towards the true rarity of the condition, and it is therefore conceivable that bilateral DCs are either underreported or underrecognised as sometimes they are known to regress spontaneously.

DCs are usually painless unless secondarily infected and may cause facial swelling only when they have reached grotesque proportions. Therefore, early detection can be challenging. Delayed eruption of teeth is also seen [3, 5, 7]. It is imperative to perform radiographic examination in cases of unerupted teeth. In fact, DCs are frequently discovered when the radiographs are taken to investigate a missing tooth, malalignment, or the failure of tooth eruption [3, 5]. A panoramic radiograph is an excellent option for this examination. The advantage of this imaging modality is that occult lesions in either of the jaws can be clearly delineated as was the case in our patient. The classical radiographic picture is that of a unilocular radiolucent lesion of various sizes with well-defined sclerotic borders associated with the crown of an unerupted tooth. This modality also helps in differentiating a normal follicular space from that of a DC. While normal follicular space is 3-4 mm, a follicular space more than 5 mm points towards the possibility of the DC [4, 5]. Displacement of adjacent teeth and resorption of teeth roots can also be observed radiographically [4, 13]. In our case, resorption was seen in the roots of the right maxillary deciduous canine and the right maxillary deciduous second molar as well as in the right maxillary permanent first premolar with displacement of the impacted right maxillary canine towards the medial aspect of the right orbital floor. Other lesions may share the same radiological features as DCs such as periapical cysts, odontogenic keratocysts, or unicystic ameloblastomas. Although involvement of the tooth, cortical expansion, and radicular resorption are characteristics more related to DCs, other lesions were not excluded until the results of pathological analysis were known. Odontogenic keratocysts do not expand the bone to the same degree as DCs and are less likely to produce teeth resorption. Clinically and radiographically, unicystic ameloblastomas and DCs cannot be differentiated, and therefore, in such cases, the histopathological analysis serves as the only means of differentiating the two lesions. In cases of extensive bony involvement and the presence of complex cystic lesions such as our case, advanced imaging in the form of CBCT or computed tomography becomes necessary. In the maxilla, DCs may be destructive, may be occupying the maxillary sinus and nasal cavities, and may encroach on the orbit as observed in our case. Advanced imaging helps in ruling out solid or fibro-osseous lesions, displays bony details, and gives the exact information about the size, origin, content, expansion of cortical plates, and relationship of lesions to adjacent anatomical structures [3, 14, 15].

Although radiographic examination provides valuable information, histopathological examination is paramount for definite diagnosis and for ruling out the possibility of other pathologies included in the differential diagnosis. The cystic lining has an inherent ability for metastatic change largely due to areas of orthokeratinisation, ciliated cells, or mucin-secreting cells present in the cystic lining. As a result of this, some DCs may progress to odontogenic keratocysts, ameloblastomas, mucoepidermoid carcinomas, or squamous cell carcinomas which are more aggressive lesions [4]. In the present case, both the cystic linings were devoid of any metaplastic or dysplastic changes.

As far as treatment is concerned, most DCs are treated with enucleation and removal of associated teeth as was advocated by us. Large DCs have also been treated by marsupialization when enucleation might otherwise result in neurosensory dysfunction or predispose to increased chances of pathological fracture. Some authors have suggested that surgery is not the only treatment modality for DC [16, 17]. Two rare cases of bilateral DCs treated with the conservative approach have been reported, both of which progressed to spontaneous resolution preventing unnecessary surgical procedures. Although, the mechanism involved in such a spontaneous resolution is still unknown. The prognosis is usually excellent, and recurrence has been nonexistent with this entity. Unfortunately, we could not comment on the prognosis as the patient was lost to follow-up immediately following discharge from the hospital.

In conclusion, the bilateral DC not associated with any syndrome or systemic disease is an extremely rare finding and therefore necessitates a special mention. Thorough clinical examination aided with systemic examination is paramount to rule out any associated syndromes or systemic diseases. Early diagnosis using both conventional and advanced imaging modalities is important to reduce morbidity and avoid more aggressive surgical procedures.
